# Five Cases of Military Surgery

**Published:** 1865-08

**Authors:** Israel B. Washburn

**Affiliations:** Late Surgeon 46th Ind. Vol. Inf.


### FIVE CASES OF MILITARY SURGERY.

BY ISRAEL B. WASHBURN, M. D.,

*Late Surgeon 46th Ind. Vol. Inf.*

*Case* 1.—Samuel Clark, private Co. F, 28th Iowa Inft. Wounded while lying in quarters during the siege of Vicksburg. The ball entered the left buttock high up, passing downward and lodging in the perineum; it was extracted and dressed as usual with water dressings. Upon the sixth day secondary homorrhage occurred. After several ineffectual attempts at ligating the artery by a "Surgeon" present, the bleeding was almost immediately suppressed by the application of persulphate of iron. I have observed that surgeons very frequently suggest and practice the heroic when the more simple remedies would be entirely successful. However, I remember an instance of a different kind. After the battle of Champion Hill, Miss., an Assistant Surgeon attending the ward containing the amputations, applied the presulphate of iron in a case of secondary hemorrhage from the *femoral artery!* The patient died. Assistant Surgeon resigned and is now engaged in the laudable employment of an Insurance Agent.

*Case* 2.—Bernard M. Minch, private Co. G, 29th Wisconsin Vols. Wounded in the right arm in the battle of Port Gibson, Miss., May 1st, 1863. The ball struck about the middle of the humerus, shivering it very badly. Resection was performed in the usual manner, and about four inches and a half of the shaft removed. The patient received a flesh wound of the right hand at the same time; both wounds healed after the operation with scarcely a bad symptom. He was discharged *well* July 17th, '63. He was able to write with the crippled hand and arm the last I heard of him, which was through Dr. D. DuBois, Surgeon 29th W. V. I., Nov. 24th, '63.

*Case* 3.—Daniel Cobb, private Co. H, 46th Ind. Vols. Wounded in the battle of Port Gibson, May 1st, '63. The right humerus was fractured in its upper third by a conical ball. Resection of the head and three inches of the shaft of the bone. The shock from the wound being very severe and reaction not being perfect, the wound did not do well, the suppuration being profuse after the fourth day until the death of the patient, which occurred the twelfth day. Instead of leaving those most severely wounded near the field, they were moved two or three times, thereby preventing the union of fractures, and causing more than one case of fatal secondary hemorrhage.

*Case* 4.—Wm. H. H. Sales, private Co. C, 34th Ind. Vols. Wounded May 23rd, '63, during the investment of Vicksburg. Fracture of the humerus. Resection—the head and three inches and a half of the shaft removed. I saw the patient the 27th day after the operation, the wound was then nearly closed, the pus and granulations healthy. He was sent to General Hospital the 34th day, at which time he thought himself "almost well." I never learned whether the limb was of any use to him or not after recovery.

*Case* 5.—Henry C. Cruger, private Co. H, 34th Ind. Vols. Wounded July 2d, '63, by accidental discharge of his musket while his fore-arm was resting on the muzzle. The radius was greatly shattered in its middle third, the muscles lacerated and powder-burnt. Resection—three inches and a half of the shaft was removed by Surgeon R. B. Jessup, 24th Ind. Vols., the ulna left intact. The wound was treated with cold water dressings and seemed to be doing very well until the third day, when the patient had a chill which every Surgeon present supposed would be followed by pyemia. Quinine was given freely, and the wound carefully syringed with dilute alcohol, which treatment was continued two days. The patient seemed entirely free of fever after that, and whisky and tincture of iron were administered instead of the quinine. A yeast poultice was applied and frequently changed, but the benefit of it was considered very doubtful, and water dressings were re-applied, the patient gradually improving.

Eighteenth day. He was sent to General Hospital, at which time he was doing well, the wound healing rapidly and the patient appearing to be entirely out of danger.

